# AI-Assisted Forensic Analysis of Hanging-Related Ligature Marks: A Pilot Study Using Convolutional Neural Networks

**DOI:** 10.3390/diagnostics16142247

**Published:** 2026-07-18

**Authors:** Giorgia Rigano, Fabrizio De Vita, Lucia Candela, Dario Bruneo, Salvatore De Caro, Marija Čaplinskienė, Elvira Ventura Spagnolo, Gennaro Baldino

**Affiliations:** 1 Department of Biomedical and Dental Sciences and Morphofunctional Imaging, University of Messina, Via Consolare Valeria, 1, 98125 Messina, Italy; giorgia.rigano@studenti.unime.it (G.R.); lucia.candela@studenti.unime.it (L.C.); eventuraspagnolo@unime.it (E.V.S.); 2 Department of Engineering, University of Messina, Contrada Di Dio, Sant’Agata, 98166 Messina, Italy; fabrizio.devita@unime.it (F.D.V.); dario.bruneo@unime.it (D.B.); salvatore.decaro@unime.it (S.D.C.); 3 State Forensic Science, Mykolas Romeris University Vilnius, 01101 Vilnius, Lithuania; m.caplinskiene@gmail.com

**Keywords:** artificial intelligence (AI), machine learning, forensic pathology, forensic science, hanging

## Abstract

**Background:** Artificial intelligence (AI) is increasingly applied in medical image analysis, although its application in forensic pathology remains limited. The assessment of ligature marks in hanging deaths is challenging and relies on forensic expertise. This pilot study evaluated a deep learning approach for morphological classification of hanging-related ligature marks. **Methods:** A Convolutional Neural Network (CNN) was trained on a dataset of 404 standardized JPEG images obtained from forensic medicine atlases and classified into hanging-related ligature marks and non-hanging lesions, including strangulation and post-mortem artefacts. Following internal validation, the model was tested on an independent set of forensic case images provided by forensic pathology experts from Messina, Italy, and Vilnius, Lithuania. Images were annotated and reviewed by a team of two forensic pathologists, with final labels assigned by consensus using morphological criteria. **Results:** The CNN demonstrated encouraging classification performance with an F1-score of 0.81 ± 0.04, distinguishing hanging-related ligature marks from morphologically similar lesions. The methodological framework and image standardization criteria for AI-assisted forensic analysis were also established. **Conclusions:** AI-based image analysis may support the evaluation of ligature marks during external examinations. Nevertheless, forensic diagnosis requires the integration of autopsy findings, physical examination, and circumstantial evidence. Larger datasets and multicenter protocols are needed to further assess reliability and applicability.

## 1. Introduction

Hanging is among the most frequently encountered forms of violent asphyxial death in forensic practice. Nevertheless, the forensic diagnosis of hanging is not always straightforward. The presence of a ligature mark on the neck is not pathognomonic of hanging, as similar lesions may also occur in ligature strangulation, post-mortem suspension, or as post-mortem artefacts. Consequently, differential diagnosis may be particularly challenging, especially when conclusions predominantly rely on external examination. The external hallmark of hanging is the ligature mark, typically appearing as a depressed area of the skin. However, its morphology may vary considerably according to several factors, including whether suspension is complete or incomplete, the physical characteristics of the ligature material, the post-mortem interval, and manipulations performed during body recovery. Most importantly, the morphological characteristics of the ligature mark alone do not establish its vitality, since similar findings may also be observed in post-mortem artefacts. In order to support the diagnosis, it is essential to assess further pathognomonic external signs, autopsy and CT findings, and scene investigation findings, including body position, characteristics and length of the ligature, relationship between the body and surrounding structures, and circumstantial information. Indeed, bodies bearing ligature marks are frequently discovered in circumstances in which the original scene has been altered or the body has been removed from suspension, potentially leading to significant diagnostic errors. Furthermore, the initial scene investigation and external examination may be performed by non-forensic specialists or first responders, potentially increasing the risk of diagnostic errors, especially when the body has been removed from suspension or the ligature device is unavailable. In forensic science, interpretation of these findings still largely depends on the expertise of highly specialized professionals, often working within a multidisciplinary framework [1]. Although expert assessment remains the cornerstone of forensic diagnosis, evaluations may differ according to individual experience and subjective interpretation. In such a scenario, Artificial Intelligence (AI) has recently emerged as a promising supportive tool, contributing to greater objectivity, consistency, and efficiency while reducing observer-dependent variability. Contemporary AI systems rely on trained computational models to analyze complex and high-dimensional data in ways that approximate human cognitive processes, enabling a wide range of applications across scientific and technical fields [2,3,4], thereby strengthening the methodological robustness of forensic analyses and potentially enhancing their acceptance in legal contexts.

The present study aimed to evaluate the feasibility of a CNN-based approach for the classification of ligature marks associated with hanging, distinguishing them from other morphologically similar conditions, including strangulation and post-mortem artefacts, and to explore the potential role of deep learning as a supportive instrument in forensic image analysis. This line of inquiry raises important considerations regarding the potential role of such technologies in forensic practice. Particularly, it prompts reflection on whether AI could ever substitute the expertise of the medical examiner, whose conclusions rely on comprehensive evaluation processes that include detailed observation, systematic documentation, morphological description, histological analysis, and comparison with established scientific literature, all informed by professional experience. Moreover, the implementation of AI in this domain requires not only high levels of statistical performance, but also transparency, external validation, and the capacity to withstand scrutiny within judicial proceedings.

## 2. Methods

### 2.1. Dataset Construction and Image Preprocessing

The dataset employed in this study was assembled from standardized JPEG images sourced from established forensic medicine atlases [5,6,7,8,9,10], case reports and case series [6,7,8,9,10,11,12,13,14,15,16,17,18,19,20,21,22,23,24,25,26,27] as well as real cases of forensic pathologists from the University of Messina (Messina, Italy) and University of Vilnius (Vilnius, Lithuania). A supervised binary classification scheme was adopted, comprising two classes: 261 images depicting ligature marks consistent with hanging (positive class, labelled Hanged) and 143 images showing marks attributable to alternative forensic aetiologies such as strangulation or post-mortem artefacts capable of simulating the macroscopic appearance of hanging (negative class, labelled Not Hanged). More specifically, the “Hanged” category comprised 93 images derived from atlases/reports and 168 images obtained from real forensic cases, whereas the “Not Hanged” category included 59 images from atlases/reports and 84 from real forensic cases. Each training sample was paired with structured morphological annotations encoding discriminative features, employed to guide labelling and support feature extraction during model optimization. Images were independently annotated and subsequently reviewed by two forensic pathologists, one from Italy and one from Lithuania, with final labels assigned by expert consensus according to predefined morphological criteria.

All images were resized to a uniform spatial resolution of 224 × 224 pixels to comply with the input requirements of the selected convolutional backbone [28,29]. Pixel values were then normalized using the standard MobileNetV2 preprocessing function, which applies a fixed, dataset-independent scaling to the range expected by the pre-trained backbone; since this transformation does not rely on any statistics estimated from the data, it cannot introduce information leakage across partitions. The full dataset was partitioned into training (70%), validation (15%), and test (15%) sets at the patient level, ensuring that all images belonging to the same patient were assigned entirely to a single set; this prevented any information from the same subject from appearing simultaneously in the training and evaluation sets. Since each patient contributes a single image to the dataset, patient-level and image-level splitting are equivalent in this study. Moreover, the split was performed using stratification by class, so that the label distribution was preserved across the three mutually exclusive subsets.

**Figure 1:**
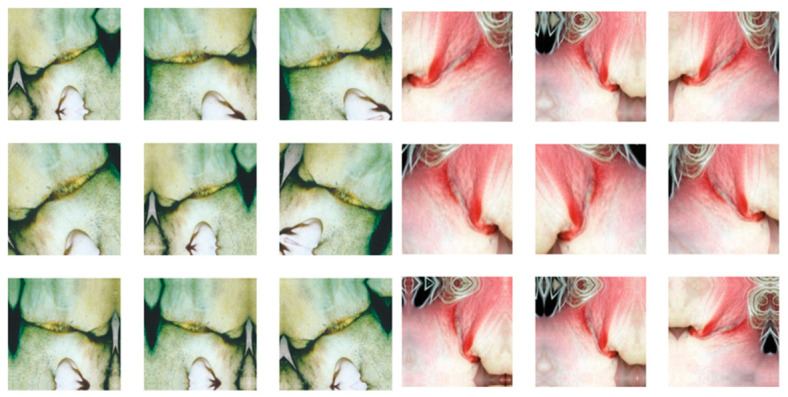
Example of augmentation strategy: images flipped, rotated and zoomed.

To mitigate the risk of overfitting and to promote model robustness in the presence of limited training data, an on-the-fly data augmentation pipeline was integrated directly into the model architecture [30,31]. The augmentation strategy comprised random horizontal flipping, random rotation (up to ±10%), and random zoom (up to ±20%), applied stochastically to each training sample at every epoch (Figure 1). It is worth mentioning that augmentation was applied exclusively to the training set, while the validation and test sets were kept in their original form, so that model selection and final evaluation were performed on unaltered data. This approach makes it possible to simulate realistic variations in acquisition conditions, such as different angles or distances, thereby artificially increasing the variability of the dataset. This is particularly important in the forensic field, where available data is often limited and not always standardized. In this way, the model has been exposed to a greater number of possible configurations, improving its robustness when analyzing images it has never seen before.

### 2.2. Model Architecture

The classification model was built upon a transfer learning paradigm leveraging MobileNetV2, a lightweight convolutional neural network pre-trained on the ImageNet dataset. MobileNetV2 was selected for its favourable trade-off between computational efficiency and representational capacity, which makes it well-suited for domains characterized by limited sample sizes. This architecture provides the best balance between predictive performance and model footprint and is, moreover, one of the most widely adopted for diagnostic purposes, as reported in several recent works [32,33,34,35]. The pre-trained convolutional base was employed as a fixed feature extractor: all its weights were frozen (i.e., set to non-trainable) so that the learned low-level and mid-level visual representations were preserved without modification during training.

**Figure 2:**
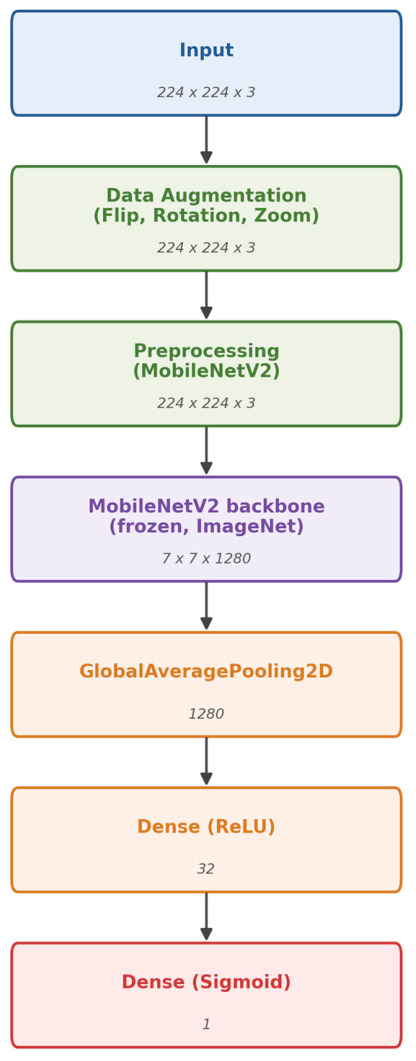
Proposed model architecture.

A custom classification head was appended on top of the frozen base and consisted of the following layers: (i) a Global Average Pooling layer, which reduced each feature map to a single scalar value, thereby decreasing the total number of parameters and mitigating overfitting; (ii) a fully connected (Dense) layer with 32 units and ReLU activation, serving as the learned decision layer; and (iii) a single-unit output layer with sigmoid activation, producing a probability estimate for the positive class. Input images were preprocessed using the MobileNetV2-specific preprocessing function, which scales pixel values to the (−1, 1) range as expected by the pre-trained weights. The proposed model shown in Figure 2 comprises 2.2 M parameters, of which only 41 K are trainable, since the MobileNetV2 backbone is kept frozen and only the classification head is fine-tuned. The resulting model occupies 8.77 MB of storage, making it suitable for deployment on devices with limited computing capabilities.

### 2.3. Training Protocol and Hyperparameters

The model was compiled with binary cross-entropy as the loss function and optimized using the Adam optimiser with a learning rate of 1 × 10−4. Training was conducted for a maximum of 500 epochs with a batch size of 32. Class imbalance represents a critical issue in this setting, since a skewed distribution biases the model toward the majority class while degrading the recall on the minority one; under such conditions, reporting accuracy alone would be misleading. To mitigate this effect, rather than modifying the dataset through resampling, a class-weighting strategy was adopted. Specifically, class weights were computed using the balanced strategy provided by scikit-learn, which assigns each class a weight inversely proportional to its frequency. In this way, misclassifications of the under-represented class are penalized more heavily in the loss function, driving the optimizer to pay proportionally more attention to the minority class without introducing synthetic samples or discarding real ones, thus preserving the original data distribution and preventing the model from developing a systematic bias towards the majority class. Two callback mechanisms were employed during training. First, an Early Stopping callback monitored the validation loss and halted training if no improvement (minimum delta = 0) was observed for 10 consecutive epochs, thereby preventing unnecessary computation and overfitting to the training data. Second, a Model Checkpoint callback saved the model weights corresponding to the lowest validation loss observed throughout training, ensuring that the final evaluated model reflected the best-performing state rather than the state at the last epoch.

### 2.4. Evaluation Protocol

To obtain robust and reliable performance estimates, the entire training and evaluation pipeline (including data splitting, augmentation, training with early stopping, and testing) was executed independently 50 times, each with a different random data partition. This repeated random sub-sampling strategy was adopted to account for the variance introduced by the relatively small dataset size and to provide a distribution-based assessment of model performance rather than a single point estimate.

For each run, the best-performing model (as determined by the checkpoint callback) was loaded and evaluated on the held-out test set. Predictions were binarized using a threshold of 0.5. Performance was quantified using four standard classification metrics computed for the positive class (Hanged): precision, recall (sensitivity), F1-score, and overall accuracy. Results are reported as mean ± standard deviation across the 50 independent runs.

### 2.5. Ethical Considerations

This study adhered to established ethical guidelines. Prior to analysis, all images were anonymized by cropping them to focus solely on the wound area and removing any details related to the subject’s identity, sex, anatomical location, or case-specific context. No personal identifiers or supplementary metadata were shared with the AI system. Consequently, the dataset was fully compliant with data protection regulations and did not pose any risk to individual privacy. All procedures were in accordance with the 1964 Helsinki declaration and its later amendments or comparable ethical standards. In compliance with Italian privacy laws D.Lgs 196/2023 and D.Lgs 101/2018 (integration of European regulations 2016/679), all data has been treated anonymously.

## 3. Results

To qualitatively assess what the convolutional backbone learns to encode, we visualized the intermediate feature maps produced by the network for two representative test images, one per class (i.e., Hanged and Not Hanged).

**Figure 3:**
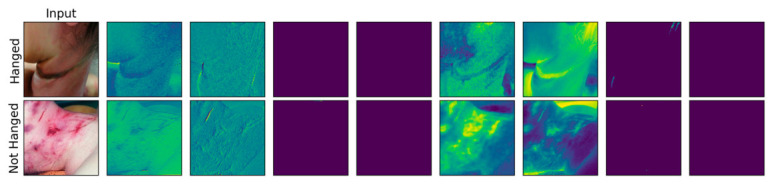
Feature maps extracted from the first convolutional layer of MobileNetV2.

Figure 3 reports the activations of the first convolutional block of the MobileNetV2 backbone, which applies a set of 3 × 3 convolutional filters with stride 2 followed by batch normalization and a ReLU non-linearity, yielding 32 feature channels at a spatial resolution of 112 × 112. For readability, only eight of the resulting channels are shown alongside the corresponding input image.

As expected for an early layer, the network responds to generic low-level visual descriptors rather than class-specific patterns. Several channels act as edge and contour detectors, producing high activations (represented by brighter) along the neck profile and the linear marks on the skin, while others capture broader texture and intensity gradients across the region of interest. A subset of channels remains almost entirely inactive: such a sparsity is a direct consequence of the ReLU non-linearity, which suppresses negative responses, and indicates that different filters specialize on different visual cues. Importantly, because the MobileNetV2 backbone is kept frozen during training, these filters correspond to the ImageNet-pretrained weights. The class discriminative adaptation to our forensic domain is therefore delegated to the trainable classification head. The fact that even these generic low-level filters already isolate the ligature marks and skin texture supports the suitability of the adopted transfer-learning strategy for this task.

The results of the 50 independent training and evaluation runs are summarized in Table 1. Across all runs, the model achieved a mean precision of 0.79 ± 0.04, a mean recall of 0.83 ± 0.07, a mean F1-score of 0.81 ± 0.04, and a mean overall accuracy of 0.74 ± 0.05.

1. Precision (0.79 ± 0.04): Precision is the proportion of true positive predictions among all instances predicted as positive. This value suggests that approximately four out of five positive predictions were correct.
2. Recall (0.83 ± 0.07): Recall (also called sensitivity or true positive rate) measures how well the model identifies actual positive cases. The model demonstrated a notably high recall, indicating a strong capability to correctly identify images depicting ligature marks consistent with hanging, thereby minimizing the rate of false negatives.
3. Accuracy (0.74 ± 0.05): Accuracy refers to the overall correctness of the model; it is the ratio of correctly predicted observations to the total observations. The overall accuracy was somewhat lower, which is expected in the presence of class imbalance and reflects the challenge of correctly classifying negative-class images that share morphological similarities with hanging-related lesions.
4. F1-Score (0.81 ± 0.04): The F1-score is the harmonic mean of precision and recall. It provides a balance between the two, and it is especially useful when one is concerned about both false positives and false negatives [36].

The variability observed across the 50 runs—with recall ranging from 0.65 to 0.97 and accuracy from 0.61 to 0.85—reflects the influence of the limited dataset size on partition-dependent performance fluctuations. Notably, the best single run achieved a precision of 0.83, a recall of 0.97, an F1-score of 0.90, and an accuracy of 0.85, suggesting that the model architecture is capable of substantially higher performance under favourable data partitions.

**Figure 4:**
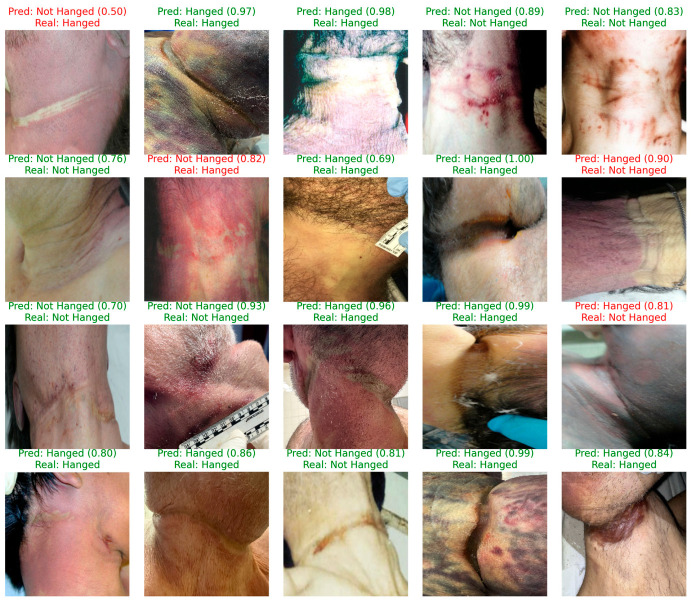
Qualitative evaluation of the proposed CNN on a representative subset of 20 images.

To provide a qualitative assessment of the classifier’s behaviour, Figure 4 shows its predictions along with the corresponding confidence on a subset of 20 images drawn from the test set. On this portion, the model produces only four misclassifications, in line with the overall performance reported in Table 1. This consistency indicates that the network has effectively learned the most discriminative morphological cues required to separate the “Hanged” and “Not Hanged” classes.

### Computational Performance and Deployment Considerations

Beyond predictive accuracy, the practicality of the proposed model for real-world forensic applications was assessed in terms of its computational requirements. All experiments were carried out on a MacBook Pro equipped with an Apple M1 Pro chip(Apple Inc. in Cupertino, CA, USA, purchased in Italy). As reported in Section 2.2, the model has a compact size of 8.77 MB. Thanks to the early-stopping mechanism, training converged on average within 50 epochs, corresponding to a total training time of approximately 200 s, while at inference the model requires only 31 ms per sample. These results confirm that the proposed model is lightweight and computationally efficient: its small footprint and low inference latency make it suitable for deployment on resource-constrained devices, supporting its practical applicability in real-world forensic scenarios.

These preliminary findings indicate that CNN-based transfer learning using MobileNetV2 can provide a meaningful baseline for the automated classification of ligature marks in a forensic context.

## 4. Discussion

The application of AI has expanded across multiple medical disciplines, with increasing interest in forensic science. To date, most applications have focused on digital forensics, where AI has shown utility in tasks such as artefact detection and evidence retrieval [37]. However, its translation to high-stakes forensic pathology remains limited due to concerns regarding data reliability, interpretability, and privacy issues. To fill this gap, the present study investigated the potential of an AI-based approach for the classification of ligature mark patterns.

Ligature marks on the neck are commonly associated with asphyxial deaths resulting from external compression of the airways. However, similar findings with some different characteristics may also be observed in strangulation and hanging, posing significant challenges in differential diagnosis, particularly when conclusions rely solely on external examination. In cases of hanging, the constriction is caused by a ligature encircling the neck and attached to a fixed point, with the tightening force generated by the weight of the suspended body. The morphological features of the ligature mark vary depending on several factors, including whether the suspension is complete or partial, the nature of the ligature material (thin and rigid versus soft and broad), the post-mortem interval prior to body discovery, and potential manipulation during resuscitation attempts [6,7,8,15].

In this context, AI was trained to analyze key morphological features of ligature marks such as their orientation (oblique or horizontal), continuity (interrupted or complete), multiplicity (single or multiple), depth uniformity, and anatomical position relative to the cricoid cartilage in order to distinguish hanging from other conditions, including post-mortem suspension, strangulation, or artefactual skin lesions.

The results of the present study demonstrate that the proposed AI-based approach has good ability to distinguish ligature marks consistent with hanging from other morphologically similar conditions, as reflected by the relatively high recall. This suggests that the model is effective in minimizing false negatives, a particularly relevant aspect in forensic contexts. However, the lower overall accuracy and the variability observed across runs highlight the challenges associated with limited datasets and the morphological similarity between different types of neck compression injuries.

Misclassifications may be attributed to several factors, including the limited dataset size, potential class imbalance, and the intrinsic morphological overlap between different types of neck compression injuries, which can challenge both human experts and AI systems.

Bodies presenting with ligature marks on the neck are frequently discovered in the absence of a forensic specialist. This circumstance can give rise to a range of interpretative errors, particularly when the body is already found on the ground, the ligature device is missing, or the body has been moved from its original position. Such factors may lead to an erroneous determination of the cause of death, including misclassification as hanging or strangulation. Such classification errors carry significant medico-legal implications: false negatives may result in missed diagnoses, whereas false positives may lead to incorrect forensic interpretations.

In this context, the availability of a support tool capable of systematically documenting the key morphological characteristics of neck marks could contribute to reducing diagnostic errors and improving the accuracy of cause-of-death assessments. This study also aims to lay the foundations for the use of a powerful technology such as CNNs in the analysis of forensic images. In this regard, the present work can be considered a proof-of-concept for the application of deep learning in forensic image classification.

However, the forensic evaluation of hanging extends beyond the analysis of ligature marks alone. For differential diagnosis, especially with cadaveric suspension, it is essential to identify vital reactions indicative of ante-mortem injury, such as hemorrhagic infiltration, skin pinching, and intra-epidermal serous vesicles [10]. Additional findings that may support the diagnosis include protrusion of the tongue clenched between the teeth, subconjunctival ecchymoses, facial congestion or pallor, hypoxemia of the extremities (glove and stocking-like distribution), and the presence of seminal fluid at the urethral meatus and/or penile erection [11]. Post-mortem examination may further reveal hemorrhagic infiltration of subcutaneous tissues and underlying musculature at the level of the ligature mark, fractures of the hyoid bone or thyroid cartilage horns, transverse intimal tears of the common carotid artery due to stretching and compression (Amussat’s sign), and, in some cases, injury to the odontoid process of the axis.

Equally important in the differential diagnosis is the integration of scene investigation findings: environmental context, spatial relationships between the body and surrounding structures, the length and positioning of the ligature, body posture, and nearby objects can provide critical insights [38]. Moreover, circumstantial evidence such as social isolation, documented psychiatric conditions, financial stressors, or interpersonal conflicts should be carefully considered [11]. The presence of additional injuries must also be interpreted cautiously, as they may result either from prior self-harm attempts or from impacts occurring during the agonal or convulsive phases [39]. Furthermore, rupture of the ligature may lead to the body being found on the ground with the noose still in place, potentially misleading a superficial examination at the scene. A key limitation of the present study is the absence of such contextual and circumstantial information, which plays a crucial role in forensic interpretation.

Moreover, despite their promising performance in image classification tasks, CNNs present several limitations that are particularly relevant in forensic image analysis. Being inherently data-hungry models, they require large-scale, well-annotated datasets to generalize robustly when trained on limited data, they tend to overfit and capture dataset-specific artefacts rather than meaningful patterns. Class imbalance may further contribute to biassed predictions and reduced generalization performance. Computational demands also remain substantial, with deep architectures requiring significant resources and processing time, limiting scalability and real-time applicability. In contrast, the lightweight design adopted in this work substantially reduces these computational demands, as demonstrated by the deployment metrics reported in Computational Performance and Deployment Considerations Section. A further constraint lies in the representation of spatial relationships: while CNNs excel at extracting local features, they may struggle with long-range dependencies and complex contextual interactions between anatomical structures. This is compounded by their well-known lack of interpretability; operating as “black-box” models, they offer limited transparency into their decision-making processes, a critical drawback in high-stakes domains such as medicine and forensics. Future developments may benefit from the integration of explainable AI techniques to improve model interpretability and support forensic validation [40,41].

Additionally, CNNs are susceptible to adversarial perturbations, where minimal and imperceptible input modifications can lead to misclassifications, and they may fail to generalize across domains due to dataset or acquisition shift. These technical limitations carry direct forensic implications: susceptibility to perturbations, opacity of reasoning, and dataset dependency collectively restrict the evidentiary reliability of CNN-based systems in legal contexts. As such, these models should not be regarded as autonomous decision-making tools, but rather as valuable support instruments during the information-gathering phase, capable of flagging relevant patterns and orienting investigative hypotheses. It is worth noting that recent advances in modern multimodal architectures, integrating visual, textual, and contextual information streams, show considerable promise in addressing several of the limitations outlined above [41]. Although such systems remain subject to errors and should not be treated as infallible, their outputs could provide valuable support to professionals in moments of uncertainty, offering an additional analytical perspective when interpretive doubt arises. Rather than being dismissed on account of their imperfections, these tools and their results should be regarded as one of the variables to be considered within the broader forensic investigation, contributing to a more informed and multi-faceted decision-making process.

## 5. Conclusions

The results of the present study highlight the potential role of CNNs as supportive tools in forensic image analysis, particularly for the classification of ligature marks associated with hanging as opposed to other morphologically similar conditions. The developed binary classification model demonstrated the feasibility of using image-based deep learning approaches to identify relevant morphological patterns and support standardized assessment of neck injuries.

The dataset was systematically divided into training, validation and hold-out test sets, and the model’s performance was evaluated through key metrics such as accuracy, precision, recall and F1-score. The obtained baseline results indicate the need for further refinement but underscore the promise of deep learning methodologies in assisting forensic professionals in the classification of injury patterns.

These findings suggest that AI-based systems may contribute to improving both the consistency and efficiency of forensic evaluations. However, their performance remains strongly dependent on dataset quality, annotation accuracy, and representativeness of the training data. Consequently, such models should currently be regarded as adjunctive instruments rather than autonomous decision-making systems.

## Figures and Tables

**Table 1:** Summary of classification performance across 50 independent runs. Values represent mean ± standard deviation for each metric. Values in brackets indicate the observed range (minimum–maximum) across all runs.

Precision	Recall	F1-Score	Accuracy
0.79 ± 0.04	0.83 ± 0.07	0.81 ± 0.04	0.74 ± 0.05
[0.68–0.89]	[0.65–0.97]	[0.71–0.90]	[0.61–0.85]

## Data Availability

The original contributions presented in this study are included in the article. Further inquiries can be directed to the corresponding author.
